# How to Make Sense of Reliability? Common Language Interpretation of Reliability and the Relation of Reliability to Effect Size

**DOI:** 10.1177/01466216251350159

**Published:** 2025-06-24

**Authors:** Jari Metsämuuronen, Timi Niemensivu

**Affiliations:** 1 Faculty of Science, Turku Research Institute for Learning Analytics, 8058 University of Turku, Turku, Finland

**Keywords:** reliability, effect size, common language effect size, common language reliability

## Abstract

Communicating the factual meaning of a particular reliability estimate is sometimes difficult. What does a specific reliability estimate of 0.80 or 0.95 mean in common language? Deflation-corrected estimates of reliability (DCER) using Somers’ *D* or Goodman–Kruskal *G* as the item-score correlations are transformed into forms where specific estimates from the family of common language effect sizes are visible. This makes it possible to communicate reliability estimates using a common language and to evaluate the magnitude of a particular reliability estimate in the same way and with the same metric as we do with effect size estimates. Using a DCER, we can say that with *k* = 40 items, if the reliability is 0.95, in 80 out of 100 random pairs of test takers from different subpopulations on all items combined, those with a higher item response will also score higher on the test. In this case, using the thresholds familiar from effect sizes, we can say that the reliability is “very high.” The transformation of the reliability estimate into a common language effect size depends on the size of the item-score association estimates and the number of items, so no closed-form equations for the transformations are given. However, relevant thresholds are provided for practical use.

## Introduction

It is sometimes difficult to communicate the precise meaning of a particular estimate of score reliability (e.g., *REL* = 0.80) to less statistically oriented people, including many researchers. In a non-statistical way, we might say that reliability “refers to the consistency of a measure” (Price et al., 2015, p. 96) or that reliability “is the consistency or repeatability of your measures” (Trochim, 2025; p. 1). In a slightly more technical way, we might say that reliability “is the degree to which measurements are free from error” (Perron & Gillespie, 2015, p. 59) or that reliability is a “measure used to quantify the amount of random measurement error present in a test score” (Metsämuuronen, 2022g, p. 1). These are not very helpful if someone wants to know what *REL* = 0.80 means.

From a technical point of view, to explain what a specific estimate of reliability means, we can refer to the traditional definition of reliability, that is,

(1) $$REL=\sigma_{T}^{2}/\sigma_{X}^{2}=1-\sigma_{E}^{2}/\sigma_{X}^{2}$$

where $\sigma_{T}^{2}$ , $\sigma_{X}^{2}$ , and $\sigma_{E}^{2}$ refer to the variances of the score variable (*X*) and the true score (*T*) and error element (*E*) (e.g., Gulliksen, 1950). Then, in the case of *REL* = 0.80, we can say that “of all the variation in the score, 80% can be attributed to the true variance and 20% to the error variance.” After that, we may need to explain that “however, the true score and the error component are unobservable phenomena related to the replications of the parallel instruments, and we have several measurement models with which we approximate the magnitude of these entities.” The burden can be extended by adding that “the error variance and, consequently, the measurement error may be radically inflated” (Metsämuuronen, 2024). So, what does the value *REL* = 0.80 actually mean?

Helpfully, and fortunately, most applied users of reliability estimates believe authorities when they refer to rules of thumb to be used as a yardstick for a reliability estimate obtained. Usually, if *REL* = 0.80, we would say that the instrument is “acceptable” and refer to the classic sources such as Nunnally (1967, 1978) or Nunnally and Bernstein (1994). We can increase the burden on the applied user by complicating the matter by noting that “the finer the distinction that needs to be made, the better the reliability must be” (Cortina, 1993, p. 101; see also the discussion in Cho & Kim, 2015; Cho & Chun, 2018). The burden can be extended even further by saying that “in many applied settings a reliability of .80 is not nearly high enough” (Nunnally, 1978, p. 245) and, if important decisions are to be made, “a reliability of .95 should be considered the desirable standard” (Nunnally, 1978, p. 246). As an aside, it should be noted that in the dataset of Hoeksta and colleagues (2019), only 16% of the authors of articles in prestigious journals were able to indicate that the lower bound of the reliability was context dependent. It is no wonder that the most commonly used estimator of reliability, coefficient alpha, has been characterized as the most misunderstood statistic (see discussion in, e.g., Cho & Kim, 2015; Cortina, 1993; Hoeksta et al., 2019; Sijtsma, 2009). From the perspective of the difficulty of communicating the factual meaning of a particular reliability estimate, we can agree that this challenge of not really understanding the meaning of reliability may not only affect coefficient alpha but also the other reliability estimators.

Is it even possible to communicate the meaning of a particular reliability estimate using a “common language”? The short answer is yes, it is possible. Inspired by McGraw and Wong’s (1992) concept of common language effect size (CLES), this article discusses ways to extend the concept to reliability estimators. Indeed, there are certain reliability estimators in the family of deflation-corrected reliability estimators (DCER; Metsämuuronen, 2022b, 2022f, 2022g) that fulfill the basic requirement of “common language estimators of reliability” (CLER): a given reliability estimate actually means something that can be explained using a common language. These estimators and related effect size estimators are discussed in this article.

The study begins with a brief discussion of the CLES estimators. Second, relevant DCERs are discussed. Third, the forms of specific DCERs are derived, where the common language elements embedded in the formulas are visible. Because several factors affect the result, such as the number of items, no closed form for the relationship between reliability and common language estimators of reliability is derived, but instead rough thresholds for practical settings are given. Exact estimates can be computed using the attached Excel-template and R code.

## Common Language Effect Sizes

CLES refers to a way of communicating the magnitude of an effect size estimate using a common language. As McGraw and Wong (1992, p. 361) put it: “The primary value of the [CLES] is that it is better than the available alternatives for communicating effect size to audiences untutored in statistics.” A well-known example of McGraw and Wong’s (1992, p. 361) communication of the CLES relates to blind dates among young adults: if the common language effect size is 0.92, this means that in any random pairing of young adult males and females, the male will be taller than the female on 92 out of 100 blind dates.

Peng and Chen (2014) have typologized traditional effect size estimators, including CLESs. The CLERs are based on two recent estimators for this family (Metsämuuronen, 2025), which generalize the interpretation of McGraw–Wong *CL* to the polytomous ordinal setting. *PHD,* or “probability of higher subpopulation dominance,” is based on Somers’ delta (*D*; Somers, 1962) and *PHG* is based on Goodman–Kruskal gamma (*G*; Goodman & Kruskal, 1954). Both *D* and *G* estimate the probability that two randomly selected cases have the same order in two variables (e.g., Metsämuuronen, 2021a; Van der Ark & Van Aert, 2015). In testing settings, they estimate the probability that in a random pair of test takers, the one with a higher item score will also receive higher test score. *PHD* and *PHG* provide an effect size interpretation for this probability. In *PHD* and *PHG*, the term “higher subpopulation” comes from the practicalities associated with rank correlations and CLES. In measurement modeling settings, when an item is dichotomous, we have two subpopulations: those who gave the “incorrect answer” (subpopulation “0”) and those who gave the “correct answer” (subpopulation “1”). In this case, the subpopulation “1” is obviously “higher” than the subpopulation “0” in terms of ranks. For items with 0–2 points, both subpopulations “1” and “2” are “higher” than the subpopulation “0,” and the subpopulation “2” is “higher” than subpopulations “0” and “1.”

*PHD* and *PHG* are defined by the means of *D* and *G* as follows:

(2) PHD=0.5×D(g|X)+0.5=0.5×D+0.5

and

(3) PHG=0.5×G(g|X)+0.5=0.5×G+0.5

where *g* refers to the item and *X* to the score variable, *D* = D(g|X) refers to Somers’ *D* directed so that “*g* given *X*” or “*X* dependent,” and *G* = G(g|X) to the Goodman–Kruskal gamma (Metsämuuronen, 2025).^1^ To compute *D* and *G*, we define the following entities:

$$C_{ij}=\underset{h<i}{\sum }\underset{k<j}{\sum }n_{hk}+\underset{h>i}{\sum }\underset{k>j}{\sum }n_{hk}$$

$$D_{ij}=\underset{h<i}{\sum }\underset{k>j}{\sum }n_{hk}+\underset{h>i}{\sum }\underset{k<j}{\sum }n_{hk}$$

$$P=\underset{i,j}{\sum }n_{ij}C_{ij}$$

(4) $$Q=\underset{i,j}{\sum }n_{ij}D_{ij}$$

where *n*_*ij*_ is the number of cases in the cell *ij* of the two-way contingency table. Using these symbols, the sample form of D(g|X) can be expressed as follows:

(5) $$D\left(g|X\right)=D=\frac{P-Q}{P+Q+2T}$$

where *T* refers to the number of tied pairs (e.g., Metsämuuronen, 2021a). In parallel, the sample form of G=G(g|X) is as follows:

(6) $$G=G\left(g|X\right)=\frac{P-Q}{P+Q}$$

Note the crucial difference between the estimators: while *D* uses all pairs to compute the probability, *G* uses only those pairs where the direction is known. In the rest of the article, the subpopulations are indexed by *l* (from “lower”) and *h* (form “higher”), and the test items are indexed by *i*.

The rationale for the estimators in Equations (2) and (3) and their relationship to the effect size originally proposed by Cureton (1956; see also Berry et al., 2018) is discussed in detail in Metsämuuronen (2025). The rationale is summarized in Supplemental Appendix 1. Equations (2) and (3) appear to be key elements in the proposed CLERs. *PHD* and *PHG* make it possible to strictly link the logic in CLESs to CLERs based on DCERs.

## Deflation-Corrected Estimators of Reliability

### Traditional Estimators of Reliability and the Challenge of Deflation

Consider a simplified, general single-factor measurement model that combines the observed values of an item (*y*_i_), a latent variable (θ), a weighting factor *wi* that links θ with *y*_i_, and the measurement error (*e*_i_):

(7) $$y_{i}=w_{i}\theta +e_{i}$$

(e.g., Metsämuuronen, 2022a, 2022b, 2022g) generalized from the traditional model (e.g., Cheng et al., 2012; McDonalds, 1999). When considering the sum of *k* independent items, Equation (7) generalizes to the following form:

(8) $$\overset{k}{\underset{i=1}{\sum }}y_{i}=\overset{k}{\underset{i=1}{\sum }}w_{i}\theta +\overset{k}{\underset{i=1}{\sum }}e_{i}$$

where assuming standardized variables and uncorrelated errors, the error variance associated with the set of the items is as follows:

(9) $$\overset{k}{\underset{i=1}{\sum }}\psi_{i}^{2}=\sigma_{E}^{2}=\overset{k}{\underset{i=1}{\sum }}\left(1-w_{i}^{2}\right)$$

which can be used to estimate the reliability of the score.

In the traditional measurement model, the weighting factor $w_{i}$ is the product-moment correlation coefficient (PMC; Pearson, 1896) in the form of item-total correlation (*Rit =* $\rho_{iX}$ ), principal component loading, or factor loading ( $\lambda_{i\theta }$ ). In the general model, $w_{i}$ is usually a correlation coefficient in *some* form, including $\lambda_{i\theta }$ . Some good options will be discussed later. In the normal cases, $-1\leq w_{i}\leq +1$ . The unobservable, theoretical latent variable θ is usually manifested as either the number-correct score (*θ*_*X*_), the principal component score (*θ*_*PC*_), the factor score (*θ*_*FA*_), a score formed by IRT modeling or Rasch modeling (*θ*_*IRT*_), or as various non-linear combinations of the items (*θ*_*Non-Linear*_).

The challenge in the traditional reliability estimators is caused by the poor behavior of PMC in the measurement modeling settings. PMC can reach the extremes of correlation (±1) only when the scales of the two variables of interest are identical (see algebraic reasons and simulations in, e.g., Metsämuuronen, 2022d, 2022c). In all other conditions, the PMC estimates underestimate the association between the variables, that is, the estimates are attenuated or deflated. This underestimation is obvious in the measurement modeling settings because the scales of the items and a score are obviously different from each other. The estimates of the PMC are particularly affected by the item difficulty: if the item difficulty is close to either the difficult or easy extreme, the PMC will approach zero, regardless of the true item-score association. This challenge is obvious in educational testing settings where we typically use items with a wide range of difficulty levels in the test (see discussion in Metsämuuronen, 2023, 2024b).

The challenge with the traditional reliability estimators is that the negatively biased PMC is embedded in the most commonly used reliability estimators such as coefficient theta (chronologically, Kaiser & Caffrey, 1965; Armor, 1974):

(10) $$\rho_{\theta }=\frac{k}{k-1}\left(1-\frac{1}{\overset{k}{\underset{i=1}{\sum }}\lambda_{i\theta }^{2}}\right)$$

and omega (Heise & Bohrnstedt, 1970; McDonald, 1970, 1999):

(11) $$\rho_{\omega }=\frac{{\left(\overset{k}{\underset{i=1}{\sum }}\lambda_{i\theta }\right)}^{2}}{{\left(\overset{k}{\underset{i=1}{\sum }}\lambda_{i\theta }\right)}^{2}+\overset{k}{\underset{i=1}{\sum }}\left(1-\lambda_{i\theta }^{2}\right)}$$

Coefficient alpha and maximal reliability are discussed in Supplemental Appendix 2.

The poor behavior of the PMC in the measurement modeling settings leads to a negative bias in the reliability estimates, especially when the test contains both easy and difficult items, as discussed above. In some cases, reliability estimates have been reported to be deflated by 0.60–0.70 reliability units (see, e.g., Gadermann et al., 2012; Metsämuuronen, 2022b, 2022f, 2022g, 2023; Zumbo et al., 2007). A negative bias of this magnitude cannot be explained by modeling error, differences between estimators, or the different score variables.

To understand the radical deflation in the correlation and reliability estimates, Metsämuuronen, 2022a, 2022b, 2022d, 2022f, 2022g has used the concept of “mechanical error in the correlation estimates” (MEC). Because of MEC, reliability estimates are attenuated or deflated, sometimes radically, depending on the type of items in the test. Deflation-corrected estimators of reliability (DCER) are shortcuts to reduce the effect of MEC in the estimates.

### DCERs Using D and G as the Weighting Factors

By replacing $\lambda_{i\theta }$ in Equations (10) and (11) with D or *G*, we obtain a variety of deflation-corrected reliability estimators based on different forms of reliability. In the main text, only DCERs based on omega and theta are discussed (see Appendix 2 for more details).

From now on, the association estimators are sub-indexed shorter by *D*_*i*_ and *G*_*i*_ when referring to a set of items. The “*omegaD*” estimator using equation (11) as the base and *D* as the linking factor is as follows:

(12) $$\rho_{\omega D}=\frac{{\left(\overset{k}{\underset{i=1}{\sum }}D_{i}\right)}^{2}}{{\left(\overset{k}{\underset{i=1}{\sum }}D_{i}\right)}^{2}+\overset{k}{\underset{i=1}{\sum }}\left(1-D_{i}^{2}\right)}$$

and “*omegaG*” using *G* as the linking factor is as follows:

(13) $$\rho_{\omega G}=\frac{{\left(\overset{k}{\underset{i=1}{\sum }}G_{i}\right)}^{2}}{{\left(\overset{k}{\underset{i=1}{\sum }}G_{i}\right)}^{2}+\overset{k}{\underset{i=1}{\sum }}\left(1-G_{i}^{2}\right)}$$

The same manner can be done also with theta-based estimators based on Equation (10): by changing the principal component loading $\lambda_{i\theta }$ , with either *D* or *G,* we obtain “*thetaD*” and “*thetaG*” as follows:

(14) $$\rho_{\theta D}=\frac{k}{k-1}\left(1-\frac{1}{\overset{k}{\underset{i=1}{\sum }}D_{i}^{2}}\right)$$

and

(15) $$\rho_{\theta G}=\frac{k}{k-1}\left(1-\frac{1}{\overset{k}{\underset{i=1}{\sum }}G_{i}^{2}}\right)$$

Of the estimators (12) to (15), those using *D* as a weighting factor are more conservative than those using *G*. Estimators based on alpha, theta, and omega are conservative and those based on rho are liberal, that is, with small sample size sizes the estimates by alpha, theta, and omega tend to slightly underestimate the population value and those by rho tend to slightly overestimate it (see Metsämuuronen, 2022g).

Metsämuuronen, 2022a, 2022b, 2022g points out that the use of theta, omega, and rho outside of their traditional context of principal component and factor analysis is debatable. However, within the paradigm of DCERs, it is assumed that these estimators *could* be used as independent estimators. Alternatively, it is possible to think that the estimates obtained by using *G* or *D* instead of the traditional *λ*_*i*θ_ could be results of renewed procedures on principal component and factor analysis (cl. ordinal theta by Zumbo and colleagues, 2007).

## Common Language Estimators of Reliability

### PHD and PHG as Indicators of Common Language Estimators

As discussed above, simple modifications of *D* and *G*, that is, *PHD* = 0.5*D* + 0.5 and *PHG* = 0.5*G* + 0.5, have an interesting practical interpretation in terms of the common language, and this can be used to communicate the content of a particular reliability estimate. In what follows, forms of reliability are derived in which this common language element is visible.

From a common language point of view, *D* and *G* can be expressed as follows (Metsämuuronen, 2025):

(16) D(g|X)=D=2×PHD−1

and

(17) G(g|X)=G=2×PHG−1

### Elements Needed in the CLERs

For the reliability estimators, the sums of the weight statistics are of interest. Then, to estimate the deflation-corrected theta or omega (see the other formulae in Supplemental Appendix 2), either the sum or the sum of squares related to *D*s, that is, $\overset{k}{\underset{i=1}{\sum }}D_{i}$ , $\overset{k}{\underset{i=1}{\sum }}D_{i}^{2}$ , and $\overset{k}{\underset{i=1}{\sum }}\left(1-D_{i}^{2}\right)$ , or *G*s are of interest (see Equations (14) to (17)). If we consider the sum of the test items, we can write because of (16):

(18) $$\overset{k}{\underset{i=1}{\sum }}D_{i}=\overset{k}{\underset{i=1}{\sum }}\left(2\times PHD_{i}-1\right)$$

and if we consider the sum of squares, we get the following form:

(19) $$\overset{k}{\underset{i=1}{\sum }}D_{i}^{2}=\overset{k}{\underset{i=1}{\sum }}{\left(2\times PHD_{i}-1\right)}^{2}$$

The corresponding forms related to *G* are equal to Equations (18) and (19) except that the element *PHD* is *PHG*.

### Practical Examples of the Common Language Estimators of Reliability

In the general case, the forms of the DCERs related to *D* and *G* and showing the common language element can be expressed as follows. Because of Equations (14) and (19), when *D* is used as the weighting factor, the CLER based on theta (*thetaD*) can be expressed as follows:

(20) $$\rho_{\theta D}=\frac{k}{k-1}\left(1-\frac{1}{\overset{k}{\underset{i=1}{\sum }}{\left(2\times PHD_{i}-1\right)}^{2}}\right)$$

The corresponding CLER using G as the weighting factor (*thetaG*) can be expressed as follows:

(21) $$\rho_{\theta G}=\frac{k}{k-1}\left(1-\frac{1}{\overset{k}{\underset{i=1}{\sum }}{\left(2\times PHG_{i}-1\right)}^{2}}\right)$$

Correspondingly, based on equations (12) and (19), the CLER based on omega and using *D* as the weighting factor (*omegaD*) can be expressed as follows:

(22) $$\rho_{\omega D}=\frac{{\left(\overset{k}{\underset{i=1}{\sum }}\left(2\times PHD_{i}-1\right)\right)}^{2}}{{\left(\overset{k}{\underset{i=1}{\sum }}\left(2\times PHD_{i}-1\right)\right)}^{2}+\overset{k}{\underset{i=1}{\sum }}\left(1-{\left(2\times PHD_{i}-1\right)}^{2}\right)}$$

and the corresponding CLER based on omega and using *G* as the weighting factor (*omegaG*) can be expressed as follows:

(23) $$\rho_{\omega G}=\frac{{\left(\overset{k}{\underset{i=1}{\sum }}\left(2\times PHG_{i}-1\right)\right)}^{2}}{{\left(\overset{k}{\underset{i=1}{\sum }}\left(2\times PHG_{i}-1\right)\right)}^{2}+\overset{k}{\underset{i=1}{\sum }}\left(1-{\left(2\times PHG_{i}-1\right)}^{2}\right)}$$

These forms in Equation (20) through (23) may not be practical for general use because it is easier to use the forms where *D* and *G* are visible (Equations (12) through (15)). The new forms are derived only to show the effect size element embedded in the formulae. The same kind of estimators can be derived based on coefficient alpha and maximal reliability (see Supplemental Appendix 2).

Although the effect size elements are embedded in the estimators, and although the common language interpretation of these elements is straightforward, their relationship to the actual reliability estimate is not straightforward because the number of items in the test is related to the outcome. This is discussed using a numerical example, and rough thresholds are provided for practical users to assess the reliability estimate in terms of effect size.

## Numerical Examples of the Relationship of Effect Size and Reliability

### Computing the DCERs for Common Language Interpretation of Reliability

To give a practical example of the common language interpretation of reliability, let us reanalyze the specific dataset from Metsämuuronen and Ukkola (2019), discussed in Metsämuuronen, 2022a, 2022b, 2022g. The dataset is based on a very simple screening test of language skills related to the language used in the test. Only those students for whom the language of instruction is a second language were expected to make mistakes and, thus, most of the test-takers were expected to score very high. Of the test-takers, 72% achieve the full score in the national sample of 7,770 students. The traditional reliability estimates are notably deflated yielding $\rho_{\alpha }$ = 0.252, $\rho_{\theta }$ = 0.444, $\rho_{\omega }$ = 0.422, and $\rho_{\mathrm{MAX}}$ = 0.492. The dataset is not published, but the main features have been discussed by Metsämuuronen, 2022a, 2022b, 2022g. Of the 8 items, 5 are binary and 3 have a 0–1–2 scale. All items appear to be very easy (0.892 < *p* < 0.992).

The calculation of DCERs and their common language interpretations are discussed here by comparing the result of different types of score variables: number-correct score ( $\theta_{X}$ ), one-parameter IRT theta score ( $\theta_{1PL}$ ), principal component score ( $\theta_{PCA}$ ), factor score based on maximum likelihood estimation by using item-wise Pearson correlations ( $\theta_{MLR}$ ) and tetrachoric/polychoric correlations ( $\theta_{MLP}$ ), and factor score based on principal axis factoring by using item-wise Pearson correlations ( $\theta_{PAF}$ ). For the sake of brevity, only the estimates based on *G* and *PHG* are discussed. The item-score correlations, associated derivatives, and reliability estimates are summarized in Table 1.

**Table 1.** Item-score correlations (G) with different score variables, relevant derivatives, and reliability estimates

	Max	*p*	X	1PL	MLR	MLP	PAF	PCA
Item 1	1	0.964	0.857	0.857	0.940	0.925	0.940	0.937
Item 2	1	0.984	0.846	0.846	0.957	0.967	0.957	0.960
Item 3	1	0.992	0.911	0.911	0.995	0.993	0.994	0.994
Item 4	1	0.909	0.834	0.834	0.892	0.879	0.892	0.892
Item 5	2	0.892	0.979	0.979	0.736	0.737	0.736	0.737
Item 6	1	0.985	0.831	0.831	0.934	0.953	0.936	0.939
Item 7	2	0.986	0.897	0.897	0.844	0.901	0.844	0.856
Item 8	2	0.990	0.924	0.924	0.993	0.986	0.992	0.984
Sum *G*			7.079	7.079	7.291	7.341	7.291	7.299
Mean *G*			0.885	0.885	0.911	0.918	0.911	0.912
Sum *G*^2^			6.283	6.283	6.698	6.785	6.697	6.709
Sum (1–*G*^2^)			1.717	1.717	1.302	1.215	1.303	1.291
*ThetaG*			0.961	0.961	0.972	0.974	0.972	0.973
*OmegaG*			0.967	0.967	0.976	0.978	0.976	0.976

max = maximum score; *p* = percentage of correct answers; X = $\theta_{X}$ , number-correct score; 1PL = $\theta_{1PL}$ , theta score from one-parameter logistic model; MLR = $\theta_{MLR}$ , factor score based on ML estimation and product-moment correlation; MLP = $\theta_{MLP}$ , factor score based on ML estimation and polychoric correlation; PAF = $\theta_{PAF}$ , factor score based on principal axis factoring; PCA = $\theta_{PCA}$ , principal component score.

The DCER based on theta and using *G* as the weighting factor and the number-correct score as the score variable ( $\theta_{X}$ ) is calculated by using Equation (15) as follows: $\rho_{\theta G\_\theta_{X}}=\frac{8}{7}\left(1-\frac{1}{6.283}\right)=0.961$ , and the corresponding omega by Equation (13) as follows: $\rho_{\omega G\_\theta_{X}}=\frac{7.079^{2}}{7.079^{2}+1.717}=0.967$ . The other estimates are calculated in parallel from the derivatives in Table 1.

Four points are worth noting. First, the estimates for scores with fewer categories ( $\theta_{X}$ and $\theta_{1PL}$ ) are less accurate than those with more categories ( $\theta_{MLR}$ , $\theta_{MLP}$ , $\theta_{PAF}$ , and $\theta_{PCA}$ ). However, the deflation-corrected reliability estimates are very close to each other (0.96–0.97 for estimates based on theta and 0.97–0.98 for those based on omega). Second, the estimates based on omega are slightly larger than those based on theta, although this can be seen in the second decimal place. Third, the estimates using the matrix of tetrachoric/polychoric correlations ( $\theta_{MLP}$ ) are slightly higher than those using the product-moment correlation ( $\theta_{MLR}$ and $\theta_{PAF}$ ). This difference is seen at the third decimal place. Fourth, the estimates for the settings using EFA and PCA are the same to 4 decimal places. Since the order of the cases does not change radically, it appears that the DCERs are very robust. Since Equation (17), each DCER in Table 1 has a common language interpretation. This is discussed below.

### Common Language Interpretation of the Reliability Estimates

**Figure 1.**
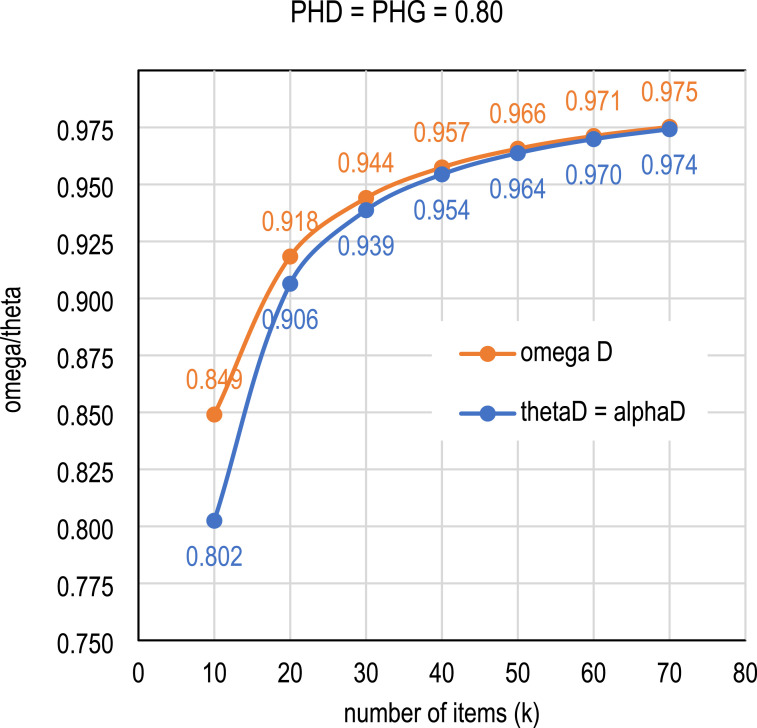
Relationship of Reliability and Length of Test When the Average Effect Size of the Items is Fixed to PHD = PHG = 0.80 (“Very Large” Effect Size).

**Figure 2.**
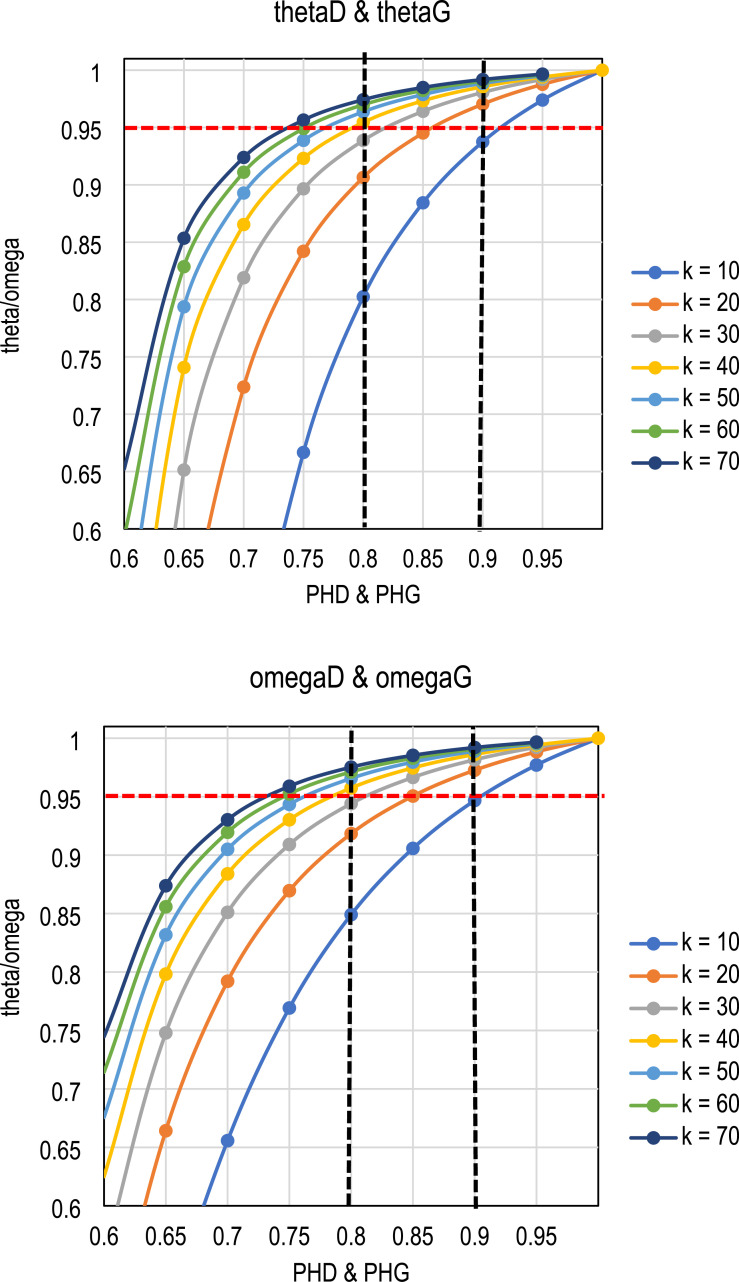
Relationship of Reliability, Effect Size, and the Number of Items in the Set thetaD and thetaG.

In general, the magnitude of the item-score correlations varies from item to item. Table 2 and Figure 1 illustrate a simplified (hypothetical) setting in which the number of items remains the same but the item-score correlations vary from perfect (*D* = *G* = 1) to very low (*D* = *G* = 0.13). More detailed benchmarks for the CLER are given in Tables 3 and 4, and Figure 2. For a more precise combinations of *k* and *D* or *G,* a spreadsheet template is included as a supplemental tool. Also included is an R package that provides more precise estimates of DCERs and corresponding CLERs.

**Table 2.** Simplified numerical thresholds of the relationship of reliability and effect size with fixed number of items in the test (*k* = 40)

*k*	Average *D* and *G*	$\overset{k}{\underset{i=1}{\sum }}D_{i}$ and $\overset{k}{\underset{i=1}{\sum }}G_{i}$	$\overset{k}{\underset{i=1}{\sum }}D_{i}^{2}$ and $\overset{k}{\underset{i=1}{\sum }}G_{i}^{2}$	$\overset{k}{\underset{i=1}{\sum }}\left(1-D_{i}^{2}\right)$ and $\overset{k}{\underset{i=1}{\sum }}\left(1-G_{i}^{2}\right)$	thetaD and thetaG Equation (22 and 23)	omegaD and omegaG Equation (24. 25)	Average PHD and PHG^a^	Effect size based on PHD and PHG^a^
40	1	40	40	0	1	1	1	Huge
40	0.9	36	32.4	7.6	0.994	0.994	0.95	Huge
40	0.8	32	25.6	14.4	0.986	0.986	0.90	Very large
40	0.7	28	19.6	20.4	0.973	0.975	0.85	Very large
40	0.6	24	14.4	25.6	0.954	0.957	0.80	Large
40	0.5	20	10.0	30.0	0.923	0.930	0.75	Large
40	0.4	16	6.4	33.6	0.865	0.884	0.70	Medium
40	0.3	12	3.6	36.4	0.741	0.798	0.65	Medium
40	0.2	8	1.6	38.4	0.385	0.625	0.60	Small
40	0.13	5.2	0.7	39.3	a	0.407	0.56	Very small

a) Reliability is negative.

^a^For the interpretation, see Table 3.

**Table 3.** Thresholds for estimates of effect sizes for binary items^a^ (Metsämuuronen, 2024a, 2025)

	Cohen’s *d*^b^	Somers’ *D*^c^	Goodman–Kruskal *G*^c^	PHD^c^	PHG^c^
Very small	0.1^d^	0.07	0.08	0.54 (0.46)	0.54 (0.46)
Small	0.2	0.13	0.14	0.57 (0.43)	0.57 (0.43)
Medium	0.5	0.29	0.31	0.64 (0.35)^e^	0.64 (0.35)^e^
Large	0.8	0.43	0.45	0.72 (0.28)	0.73 (0.27)
Very large	1.2^d^	0.59	0.62	0.80 (0.20)	0.81 (0.19)
Huge	2^d^	0.81	0.84	0.91 (0.09)^f^	1.93 (0.07)

^a^In polytomous settings, the threshold depends on the number of the subpopulations in *g* and the discrepancy in the proportions of subpopulations (Metsämuuronen, 2024a).

^b^Cohen (1988).

^c^Based on *n* = 7,948 estimates from empirical datasets by Metsämuuronen (2022d, 2025) and a quadratic model.

^d^Sawilowsky (2009).

^e^Based on modeling with Greiner’s relation and empirical dataset (Metsämuuronen, 2024a). For the quadratic model, the threshold is 0.65. Otherwise, the quadratic model and Greiner’s relation give identical outcome.

^f^PHD = 0.91 refers to 91 pairs out of 100, and PHD = 0.09 refers to 9 pairs out of 100. Both are equally rare cases and refer to “huge” in terms of effect size.

**Table 4.** Correspondence of thresholds for estimates of common language estimates of reliability and common language effect sizes

Number of items k	Mean of Ds or Gs	thetaD/G ≈ alphaD/G	omegaD/G ≈ rhoD/G	PHD or PHG	Effect size (PHD)	Effect size (PHG)	Number of items k	Mean of Ds or Gs	thetaD/G ≈ alphaD/G	omegaD/G ≈ rhoD/G	PHD or PHG	Effect size (PHD)	Effect size (PHG)
10	1	1	1	1	Huge	Huge	10	0.5	0.67	0.77	0.75	Large	Large
30	1	1	1	1	Huge	Huge	20	0.5	0.84	0.87	0.75	Large	Large
50	1	1	1	1	Huge	Huge	30	0.5	0.90	0.91	0.75	Large	Large
10	0.9	0.974	0.977	0.95	Huge	Huge	40	0.5	0.92	0.93	0.75	Large	Large
20	0.9	0.988	0.988	0.95	Huge	Huge	50	0.5	0.94	0.94	0.75	Large	Large
30	0.9	0.992	0.992	0.95	Huge	Huge	60	0.5	0.95	0.95	0.75	Large	Large
40	0.9	0.994	0.994	0.95	Huge	Huge	10	0.4	0.42	0.66	0.70	Medium	Medium
50	0.9	0.995	0.995	0.95	Huge	Huge	20	0.4	0.72	0.79	0.70	Medium	Medium
60	0.9	0.996	0.996	0.95	Huge	Huge	30	0.4	0.82	0.85	0.70	Medium	Medium
10	0.8	0.94	0.95	0.90	Very large	Very large	40	0.4	0.87	0.88	0.70	Medium	Medium
20	0.8	0.97	0.97	0.90	Very large	Very large	50	0.4	0.89	0.90	0.70	Medium	Medium
30	0.8	0.98	0.98	0.90	Very large	Very large	60	0.4	0.91	0.92	0.70	Medium	Medium
40	0.8	0.99	0.99	0.90	Very large	Very large	10	0.3	a	0.50	0.65	Medium	Medium
50	0.8	0.99	0.99	0.90	Very large	Very large	20	0.3	0.47	0.66	0.65	Medium	Medium
60	0.8	0.99	0.99	0.90	Very large	Very large	30	0.3	0.65	0.75	0.65	Medium	Medium
10	0.7	0.88	0.91	0.85	Very large	Very large	40	0.3	0.74	0.80	0.65	Medium	Medium
20	0.7	0.95	0.95	0.85	Very large	Very large	50	0.3	0.79	0.83	0.65	Medium	Medium
30	0.7	0.96	0.97	0.85	Very large	Very large	60	0.3	0.83	0.86	0.65	Medium	Medium
40	0.7	0.97	0.97	0.85	Very large	Very large	10	0.2	a	0.29	0.60	Small	Small
50	0.7	0.98	0.98	0.85	Very large	Very large	20	0.2	a	0.45	0.60	Small	Small
60	0.7	0.98	0.98	0.85	Very large	Very large	30	0.2	0.17	0.56	0.60	Small	Small
10	0.6	0.80	0.85	0.80	Very large	Large	40	0.2	0.38	0.63	0.60	Small	Small
20	0.6	0.91	0.92	0.80	Very large	Large	50	0.2	0.51	0.68	0.60	Small	Small
30	0.6	0.94	0.94	0.80	Very large	Large	60	0.2	0.59	0.71	0.60	Small	Small
40	0.6	0.95	0.96	0.80	Very large	Large	40	0.13	a	0.41	0.565	Very small	Very small
50	0.6	0.96	0.97	0.80	Very large	Large	50	0.13	a	0.46	0.565	Very small	Very small
60	0.6	0.97	0.97	0.80	Very large	Large	60	0.13	0.01	0.51	0.565	Very small	Very small

a) Reliability <0.0.

Three general observations are made from Tables 2 and 4 and Figures 1 and 2 before the common language interpretation of the reliabilities in Table 1 is given. First, the magnitude of the item-score correlations estimated by *D* or *G* has a strict effect on the reliability estimate and the effect size of the estimate: the higher the item-score correlations, the higher the magnitude of the reliability estimate and the effect size.

Second, however, if the item-score correlations are fixed, test length does not affect the CLES interpretation of the score but it does affect the reliability estimates (see Table 3). That is, if the item-score correlations by *D* or *G* are at the same level, even a short test with lower reliability will have the same effect size interpretation as a longer test with higher reliability. The reason for this unintuitive phenomenon is strictly related to the difference between the effect size and reliability formulas. From an effect size point of view, it does not matter how many items we have in the test: if the item-total correlations do not increase, the interpretation remains the same.

Third, in order to obtain a “very large” effect size, the magnitude of the DCER based on omega must be higher than the DCER based on theta; the difference may be noticeable for shorter tests. The reason for this phenomenon is that the form of omega as an estimator tends to use the item-wise information more effectively than the form of theta when it comes to estimating reliability, especially with small sample sizes. Thus, if we have 10 items with *D* or *G* at the level of 0.60, the estimate by omega would be higher than by theta. However, from the viewpoint of CL interpretation, there is no difference between the estimates: both are at the level of mean *PHD* = mean *PHG* = 0.80 because the embedded *D* or *G* is equal in the estimators (see Figure 2). Then, what is “large” reliability in the theta-based estimates is not yet “large” in the omega-based estimates if the item-score correlations were the same.

### Further Elaborations of the CL Interpretation of the Reliability Estimates

When it comes to Table 1 and the common language interpretation of the reliability estimates, we can approach the result from four perspectives. First, the mean *G* varies 0.88 < *G* <0.92 depending on the score variable. Although Table 4 does not include the estimate for 8 items, we get a rough idea that if the mean *G* is around 0.90 or higher, the effect size associated with the reliability would be “huge,” regardless of the number of items in the test, the form of the score variable, and of the reliability estimate. The same interpretation would result from knowing the reliability estimates (0.961–0.976).

Second, using the supplemented Excel template, after changing the number of items (8) and the mean *G* = 0.88 (referring to the number-correct score *X*) in the template, the mean *PHG* = 0.94 indicates a “huge” effect size. With the mean *G* = 0.92 (referring to the factor score with polychoric correlations MLP), the *PHG* = 0.96, also indicating a “huge” effect size. Then, from a common language interpretation point of view, of all pairs of cases from different item categories in all items averaged, depending on the score type, we expect to see 94–96% of such pairs where the test taker who has a higher item response also achieves the higher test score. Note that this interpretation cannot be made from the original reliability estimates—DCERs are required.

Third, let us take another look at the unintuitive difference between reliability and effect size. Consider a 40-item test and the traditional threshold for a highly discriminating test of *REL* = 0.95. From Table 4 we know that if we use theta as the base and Somers’ *D* as the weighting factor to achieve *thetaD* = 0.95, we need the mean *D* ≥0.60 which gives *PHD* ≥0.6 × 0.5 + 0.5= 0.80. In terms of common language interpretation, this refers to a pattern in which 80 out of 100 random pairs of test takers from different subpopulations, those with a higher item response also has a higher test score. This can be described as a “very large” effect size or “very high” reliability (Table 3).

We can look at the phenomenon from the opposite perspective, which gives us even more unintuitive view to the relation of reliability and effect size. Suppose we need a highly discriminating test for important decisions, where 95% of the random pairs in all the items combined would be patterned such that those with a higher item response would also have higher test score, that is, where the effect size is “huge.” Then, if we use only 10 items, we need a reliability of *thetaD* = 0.974 and if we use 40 items, we need a reliability of *thetaD* = 0.994 (see Table 4). The thresholds do not change much if we use omega as the basis: if we use only 10 items, we need a reliability of *omegaD* = 0.977 and if we use 40 items, we need a reliability of *omegaD* = 0.994. Again, the explanation is that the effect size is determined by the item-score correlations; if the mean of the item–score correlations does not change, a longer test will automatically lead to higher reliability, but the CL interpretation remains intact.

Fourth, it is worth recalling that the interpretations given above for linking the reliability and effect size are based on linking the common language effect size to the epithets of the thresholds given to the Cohen’s *d*. These benchmarks have been criticized for good reasons: the epithets such as “small,” “medium,” and “large” are in many ways underdefined (see, e.g., Correll et al., 2020; Funder & Ozer, 2016; Gignac & Szadorai, 2016). Cohen himself was well aware of this challenge: the labels are “*relative not only to each other but also to the field of behavioral science or, more specifically, to the specific content and research method employed in the particular investigation*” (Cohen, 1988, p. 25). Because the interpretation of thresholds may differ in different fields of research, it is possible that the thresholds for “large,” “very large,” or “huge” in the psychometric testing may differ from those in, for example, the medical sciences. This under-definition in Cohen’s original formulations was the reason why Metsämuuronen (2024a) derived the common language interpretation for Cohen’s *d* in order to unify the interpretation of effect sizes *r*, *d,* and *f*: although the interpretation of “large” to “huge” may change from domain to domain, the interpretation of “80 pairs out of 100” remains the same.

## Discussion

### Main Results in Nutshell

The starting point of the article was the observation that it is not easy to communicate the exact meaning of a reliability estimate using traditional reliability estimators. Specific deflation-corrected effect size estimators based on Somers’ *D* (*PHD*) and Goodman–Kruskal *G* (*PHG*) make it possible to link the reliability estimate to the common language effect sizes. The latter can be communicated using terms that refer to pairs of observations, such as “those who gave a correct answer” versus “those who gave an incorrect answer.” For example, the magnitude of a common language effect size of 0.80 simply means that in 80 out of 100 random pairs of test takers on a given item, those who gave the correct answer (or a higher item response) also scored higher on the test.

The forms of common language reliability estimators have been derived, although they do not necessarily need to be used in real life situations. It is easier to use the forms where the embedded association estimator *D* or *G* is visible. The derived forms only show that the effect size element is embedded in the estimators. For example, using a common language estimator of reliability, we can say that if the estimate of the reliability is *ThetaD* = 0.95 or *OmegaD* = 0.96 and we have 40 items, in 80 out of 100 random pairs of test takers from different subpopulations, those with a higher item response also score higher on the test. The thresholds depend mainly on the strength of the item-score association through *D* or *G,* and to some extent on the number of items in the test. Therefore, modeling the exact thresholds is not obvious. Rough boundaries have been given by using the average *D* or *G* over all items as a benchmark.

### Reasons Behind the Unintuitive Phenomenon

By linking the magnitude of the reliability estimate to the established effect size thresholds, it is possible to assess whether the reliability estimate is “large” or “high,” “very large” or “very high,” or even “huge,” from a different perspective than we traditionally do. Unlike with reliability, the test length does not affect the effect size of the reliability when the item-score correlations are fixed. The reason for this unintuitive phenomenon is that it does not matter how many items we have in the test, if the item-total correlations do not increase, the interpretation of the effect size remains the same.

Another unintuitive phenomenon related to the common language interpretation of reliability is that the estimates based on omega and using *G* as the weighting factor (*omegaG*) should be slightly higher to achieve a given level of effect size compared to an estimator based on theta and using *D* as the weighting factor (*thetaD*). The difference between the common language interpretations of *G* and *D* stems strictly from the empirical and theoretical models linking Cohen’s *d* with *G* and *D*. Since *G* estimates are (almost) always higher than those the *D* estimates, what might be considered “large” or “high” by *D* is not yet “large” or “high” by *G.* This also applies to reliability estimates. Essentially, the effect size depends mainly on the magnitude of *D* and *G* and not that much of the base of the formula used nor the number of items in the test. However, the estimators based on omega seem somewhat more effective in taking into account the differences in *D* and *G* than the estimators based on theta. If we widen the discussion to alpha and rho, we know that the estimates by theta are higher than those by alpha; theta formula maximizes alpha (Greene & Carmines, 1980), and estimates by rho are higher than those by omega (Cheng et al., 2012; see the discussion in Metsämuuronen, 2022g). Thus, because first, the omega form is more effective in respect to theta form in utilizing the item-wise information the estimates using omega tend to be larger than those using theta, and second, because the estimates using *G* are larger than those using *D*, what might be considered “large” or “high” by *thetaD* is not yet “large” or “high” by *omegaG.*

### Practical Applications of the Results

The procedure for using the information in Table 4 or the Supplementary Excel file for computing the common language interpretation of the DCERs is as follows:

(1) Calculate a score variable *X* (e.g., a number-correct score, a principal component score, a factor score, or an IRT score, i.e., a theta value). The more categories the score has, the more accurately we can distinguish between test takers. However, the differences between the results are subtle regardless of the score and the method used to create the score variable. Because DCERs and CLERs are robust, any of the scores formed by any of the abovementioned methods would give (roughly) the same interpretation.
(2) Compute item-total correlations between the items and the formed score variable using Somers’ *D*(*g*|*X*), that is, *D* directed so that it is “*X* dependent,” or Goodman–Kruskal *G.* Until better algorithms are developed, this serves as a shortcut to deflation-corrected reliability estimates. Alternatively, compute the average of all *D*s or *G*s associated with the score.
(3) Use the formulae (12) through (15) for deflation-corrected theta or omega, or equivalent formulae based on the alpha or rho form from Supplemental Appendix 2, to obtain a deflation-corrected reliability estimate.
(4) Take Table 4 and select the row with the closest resemblance between *k* (= number of items) and the average *D* or *G* or with the reliability estimate. Table 4 gives a rough estimate for the deflation-corrected theta and omega. The attached R code and Excel file provide more precise estimates.
(5) Based on the average *D* or *G*, the “*PHD* or *PHG*” column in Table 4 shows the rough average common language effect size of the items with that level of reliability. For example, *PHD* or *PHG* = 0.80 means that out of 100 pairs of test takers with different item response categories on all items in the test, 80 are such pairs where the test taker with the higher item responses (or correct responses in a binary item) scores higher on the test.
(6) The “effect size” column gives a verbal description of the effect size using the epithets given for Cohen’s *d* (see Table 3). The boundaries are based on the relation of *PHD* and *PHG* with Cohen’s *d* so that if *PHG* ≥0.93 or *PHG* ≥0.91 it is labeled “large” and if 0.81 ≤ *PHG* <0.93 or 0.80 ≤ *PHD* <0.91 it is labeled “very large.” Softer boundaries could also be used, and these can be adjusted using the attached spreadsheet template and R code.

### Known Limitations of the Study

An obvious shortcoming of the treatment is that no closed formulae were given for practical users of the thresholds to convert the reliability estimate to the effect size or common language values. Instead, the practical user has to approximate a rough value from a table. Two tools were provided for this: a rough tool using a common spreadsheet software and a more specific R code. Another shortcoming is that no limits were given for the coefficients alpha and rho; these are not obvious to give because they depend either on the item variances (alpha) or on the item wise *D*^2^ (theta) and (1–*D*^2^) (omega and rho). However, the limits given for theta give rough limits for alpha; the closer the item variances are to each other, the better the approximation. Rough limits for rho could be obtained from those for omega; the closer the weights are to each other the better the approximation. Even in this form, however, the reader gets an idea of how to communicate the results to a less statistically oriented audience. Systematic studies of the bounds would be beneficial.

### Final Notes

At the beginning of this article, we asked what *REL* = 0.80 really means. We now know that it is possible to be expressed in terms of common language. This interpretation depends mainly on 1) what the average of the item-total correlations of all items is, estimated by using Somers’ *D* or Goodman–Kruskal *G*. Less important, but related, are 2) what reliability estimator we used, 3) how the score variable was formed, and 4) how many items we had in your test. Based on Table 4, assuming we used the estimator based on theta, we had 10 items, and the average *D* or *G* is 0.60, the reliability would be *REL* = 0.80. The common language interpretation of this is that out of 100 random pairs of test takers with different item responses, 80 would be such that the test taker with the higher item response would also score higher on the test. This would be considered “large” or “high” effect size. However, if the average *D* or *G* were 0.30, we would need 50 items to achieve the same reliability *REL* = 0.80, and still then the effect size would be “medium” because the values of *D* or *G* are low. The reason for the different interpretation of the same size of reliability level is that the effect size is strictly related to the size of the estimates of *D* and *G*.

Note that for the effect size of the discriminative power of the test score alone, we do not need the reliability estimate. The common language effect size is calculated by using the Somers *D* or Goodman–Kruskal *G* alone without knowing the reliability estimates. It is now known that the effect size estimate is more stable than the reliability estimate; regardless of the length of the test and the base of the reliability estimator, the effect size and its CL interpretation remain intact if the item-score correlations (*D* or *G*) remain intact.

As an idea for further studies, it might be worthwhile to further investigate the embedded effect size indicators (*PHD* and *PHG*) as independent indicators of “reliability.” A relevant statistic could then be the mean of the *PHD*s and *PHG*s over all items in the set. This type of estimator could strictly indicate the effect size of the accuracy of the test score.

## Supplemental Material

Supplemental Material - How to Make Sense of Reliability? Common Language Interpretation of Reliability and the Relation of Reliability to Effect Size Supplemental Material for How to Make Sense of Reliability? Common Language Interpretation of Reliability and the Relation of Reliability to Effect Size by Jari Metsämuuronen and Timi Niemensivu in Applied Psychological Measurement

## Ethical Statement

All necessary support and approvals are in place for the research.

## Data Availability

The dataset used in the empirical section of the article is available in CSV format at https://doi.org/10.13140/RG.2.2.10530.76482 and in IBM SPSS format at https://doi.org/10.13140/RG.2.2.17594.72641 (Metsämuuronen, 2021b, 2022e).

## Note

1. Metsämuuronen (2021a) showed that *G* has a directional nature although it is often taken as a symmetric coefficient in the same way as the Pearson product-moment correlation coefficient. However, in the case of no tied pairs, such as in the case where all the test takers receive a unique score, except in the case of two continuous variables, *G* is clearly a directional coefficient, such that G=G(g|X)=D(g|X)≠D(X|g)≠D(Sym) .
