# Supplementary material for: How to Make Sense of Reliability? Common Language Interpretation of Reliability and the Relation of Reliability to Effect Size
Source: Appl Psychol Meas. 2025 Jun 24;49(7):396–416. doi: 10.1177/01466216251350159 (PMC12187714; doi:10.1177/01466216251350159)
Supplement: Supplemental Material - How to Make Sense of Reliability? Common Language Interpretation of Reliability and the Relation of Reliability to Effect Size [file sj-zip-1-apm-10.1177_01466216251350159.zip › Common language estimators of reliability 2025 APM apnds1-2 edit5.pdf]

Supplementary Appendix 1

Rationale of *PHD* and *PHG*

Metsämuuronen (2025) proposes two new estimators of common language effect size (CLES), *PHD* (“probability of higher subgroup dominance based on Somers’ delta”) (*D*; Somers, 1962) and *PHG* based on Goodman–Kruskal gamma (*G*; Goodman & Kruskal, 1954). *PHD* and *PHG* are defined as follows:

$$PHD = 0.5 \times D\left(g \middle| X\right) + 0.5 = 0.5 \times D + 0.5 \qquad (1)$$

and

$$PHG = 0.5 \times G\left(g \middle| X\right) + 0.5 = 0.5 \times G + 0.5 \qquad (2)$$

(Metsämuuronen, 2025) where *D* refers to Somers’ *D* directed so that “*g* given *X*” or “*X* dependent”, $D\left(g \middle| X\right) = D$. Because *G* appears to be a directional statistic to the same direction as $D\left(g \middle| X\right)$ (see Metsämuuronen, 2021b), it can be expressed the same manner as *D*: $G = G\left(g \middle| X\right)$. The rationale of these estimators and their connection to the effect size discussed by Cureton (1956; see also Berry, Johnson, & Mielke, 2018; Kerby, 2014) is discussed in detail in Metsämuuronen (2025). The rationale is condensed in what follows.

Effect size as proportion of favorable cases

The basic idea in Cureton’s estimator of effect size for binary settings is based on the proportions of favorable cases (*f*) and unfavorable cases (*u*): $ES = f - u = f - (1 - f) = 2f - 1$. In the item analysis settings with binary items, the favorable cases are those where, after the item responses are ordered by the score, the test takers with a correct answer (subpopulation 1) are located higher in rank in comparison with those who gave an incorrect answer (subpopulation 0). This idea relates with the original idea of Mann–Whitney *U* statistics (Mann & Whitney, 1947) related to the higher of the subpopulations (U_1) which is usually computed by using Wilcoxon (1945) statistics for the same purpose:

$$U_1 = R_1 - \left(\frac{n_1 \left(n_1 + 1\right)}{2} \right). \qquad (3)$$

where R_1 refers to the rank sum of the scores related to the subpopulation 1, ranked among all (n_1+n_2) scores, that is, Wilcoxon statistic W_1 (Wilcoxon, 1945) related to the higher of the subpopulations 0 and 1. U_1 refers to the number of pairs of observations where the cases in subpopulation 1 are located higher than the cases from the sub-population 0 after they are ordered by the metric variable (Mann & Whitney, 1947). When U_1 is proportioned with the number of all the possible pairs ($n_0 \times n_1$), we get the proportion of favorable cases of all the cases:

$$f = \frac{U_1}{n_0 n_1}. \qquad (4)$$

This statistic, as being probability, ranges 0–1. When doubled and relocated, Cureton's effect size turns to be the rank-biserial correlation (R_{RB}; see Berry, Johnston, & Mielke, 2018; Metsämuuronen, 2022e):

$$R_{RB} = 2 \times \frac{U_1}{n_0 n_1} - 1 \quad (5)$$

ranging −1 to +1. The same outcome can be obtained if using Wendt's (1976) modification of the rank-biserial correlation:

$$R_{RB} = 1 - 2 \times \frac{U_0}{n_0 n_1} \quad (6)$$

where U_0 refers to the *lower* of the subpopulations 0 and 1.

Somers' D as a base for effect size

Newson (2008) showed that Cureton's R_{RB} in Eqs. (5) and (6) is a special case of Somers' *D* restricted for the dichotomous settings and—when denoting the item by *g* and the score by *X*—directed so that "*X* dependent" or "*g* given *X*", that is, $R_{RB} = D(g|X)$ (see the rational for the unconventional notation in Metsämuuronen, 2020a, 2020b, 2021a). From this onwards, $D(g|X)$ = *D*. Metsämuuronen (2021b, 2022e) generalized the same for the polytomous settings including binary setting as a special case. This is a useful extension from the common langue estimators of reliability viewpoint, and it is discussed in what follows.

Jonckheere–Terpstra test statistics (*JT;* Jonckheere 1954; Terpstra 1952) extends the directional U_1 and its calculation procedure to polytomous cases (e.g., Siegel & Castellan, 1988). Let us assume two random variables: a categorical variable with a binary or ordinal polytomous scale (*g*) and a metric variable with an ordinal, interval, semi-continuous, or continuous scale (*X*). The observed values in *g* are denoted by y_i with $R = 1, \ldots, R$ categories referring to subpopulations and the observed values in *X* are denoted by x_j with $C = 1, \ldots, C$ categories, and $R << C$.

Let us denote the subpopulations by *l* (from "lower") and *h* (from "higher") and $l < h$. Then, by using the same logic and naming as in Cureton's effect size (Eq. 3), the following measure is rank–*poly*serial correlation (ρ_{RP}):

$$\rho_{RP} = 2 \times \frac{JT_{gX}^{Obs}}{JT_{gX}^{Max}} - 1 = 2 \times \frac{JT}{\sum_{l<h}^{R} n_l n_h} - 1 = 2 \times \sum_{l<h}^{R} U_{lh} \Big/ \sum_{l<h}^{R} n_l n_h - 1 = D(g|X) = D \quad (7)$$

(Metsämuuronen, 2021b, 2022e), where JT_{gX}^{Max} is the maximal *JT* statistics and JT_{gX}^{Obs} is the observed *JT* statistics, i.e., the sum of all *U* statistics obtained between the directional subpopulations $l < h$. Notably, the element embedded in Eq. (7),

$$\frac{JT}{\sum_{l<h}^{R} n_l n_h} = f_D = 0.5 \times D + 0.5 = PHD \tag{8}$$

(Metsämuuronen, 2025), i.e., "probability for higher subgroup dominance based on Somers' *D*", *PHD*, refers to the proportion of "favorable" cases the same manner as in Cureton's measure of effect size for binary settings. Here, the favorable cases are sub-indexed by *D* to make difference with the corresponding element based on *G* discussed later. In the special case of binary *g*, $l = 0$, and $h = 1$, and $JT = U_{01} = U_1$. Then, because of Eqs. (5) and (6), Eq. (7) reduces to

$$2 \times \frac{U_1}{n_0 n_1} - 1 = 1 - 2 \times \frac{U_0}{n_0 n_1} = \rho_{RB} = D\left(g|X\right) = D. \tag{9}$$

Eqs. (7) and (9) mean that, in the in binary settings, Somers' *D* is a rank-biserial correlation, and in ordinal polytomous settings it is a rank-polyserial correlation. Because of Eq. (8), in the measurement modelling settings, *PHD* strictly indicates the proportion of the ascending located observations in the item after they are ordered by score. If the rank-biserial (Eq. 9) or rank-polyserial correlation (Eq. 7) between the item and score appears to be $D = 0.80$, $PHD = 0.5 \times 0.8 + 0.5 = 0.90$. By using common language, from the viewpoint of "probability of higher subgroup dominance", we infer that in 90 cases out of 100 random pairs of test takers from different subpopulations, the case from a higher subpopulation scored higher in the test than the case from a lower subpopulation. In binary setting, we can say that in 90 cases out of 100 random pairs of test takers giving either correct or incorrect answer, the test taker who gave the correct answer scored higher in the test. Eq. (8) is a key element for the common language estimators of reliability (CLER) discussed in the main article.

Goodman–Kruskal G as a base for effect size

Somers' *D* and Goodman–Kruskal *G* are closely related indices for rank-order correlation in general and for item–score association in specific. With Kendall's tau-a and tau-b (Kendall, 1938, 1948), they all estimate the probability that two randomly chosen pairs in two variables have the same order (see Metsämuuronen, 2021b; Van der Ark & Van Aert, 2015). We may call this family of estimators either the tau family (e.g., Kendall, 1948; Kendall & Gibbons, 1990), gamma family (e.g., Van der Ark & Van Aert, 2015; Woods, 2007), or delta family (e.g., Newson, 2006; Metsämuuronen, 2020a, 2020b). If the variables are continuous, all these estimators equal with tau-a (see Kendall & Gibbons, 1990; Newson, 2006).

Metsämuuronen (2021b) showed that *G* has a hidden directional nature (cl., e.g., IBM, 2017; Sheskin, 2111; Sirkin, 2006; Wholey, Hatry, & Newcomer, 2015 where *G* is taken as a symmetric measure). Namely, if there are no tied pairs (*T*), that is, pairs of which we do not know the direction,

$$G = D\left(g|X\right) \neq D\left(X|g\right) \neq D\left(Symmetric\right). \tag{10}$$

Therefore, we could express *G* as $G\left(g|X\right)$ (see Eq. 3). If there *are* tied pairs, the estimates by *G* are more liberal than the ones by *D*:

$$\hat{G} = \hat{G}\left(g|X\right) > \hat{D}\left(g|X\right) = \hat{D}. \tag{11}$$

The reason for the liberal nature in *G* is that, while *D* uses *all* the pairs ($\sum_{l<h}^{R} n_l n_h$) as the base for the probability, *G* omits the tied pairs, that is, *G* uses only those pairs *where the direction is known* ($\sum_{l<h}^{R} n_l n_h - T$). (cl. the formulae of *D* and *G* in, e.g., Metsämuuronen 2021b). Although some researchers have interpreted that the estimates by *G* are "inflated" (see, e.g., Higham & Higham, 2019; Kvålseth, 2017; Masson & Rotello, 2009), the same logic of using only relevant cases is used in such procedures as the sign test and Wilcoxon signed-rank test too (see, e.g., Siegel & Castellan, 1988).

If we use only those cases where the direction is known as the base for the probability, Eq. (9) gets the form

$$\frac{JT}{\sum_{l<h}^{R} n_l n_h - T} = f_G = 0.5 \times G + 0.5 = PHG \qquad (12)$$

(Metsämuuronen, 2025; see Eq. 3). The statistics *PHG* = $0.5 \times G + 0.5$ indicates, in the same manner as Eqs. (8) and (10), the "probability of higher subgroup dominance" with the small difference that the probability is computed by using only relevant pairs. Hence, if *G* = 0.80, *PHG* equals 0.5×0.8 + 0.5 = 0.90. By using common language, we infer that in 90 out of 100 random pairs of test takers from different subpopulations, the test taker from a higher subpopulation scored higher in the test, if we consider only the cases where the scores were not equal. In binary setting, we may say that in 90 out of 100 random pairs of test takers giving either a correct or an incorrect answer, the test taker who gave the correct answer scored higher in the test, if either of the test takers had a higher score.

Limits of PHD and PHG

Metsämuuronen (2025) has offered limits of *PHD* and *PHG* comparable with the commonly used Cohen's *d* effect size; these are collected in Table 1. The boundaries are based on published empirical datasets of *n* = 7,948 estimates of effect sizes related to measurement modelling settings (Metsämuuronen, 2022f, 2023). In Table 1, we note that the tabled thresholds for Cohen's *d, D*, and *G* are absolute values; the negative value indicates that the cases from lower group(s) were superior to the cases in the higher group(s). In contrast, as being probabilities, the thresholds for *PHD* and *PHG* are non-negative. Then, because of symmetricity, also the low probabilities are relevant from the effect size viewpoint. That is, while 80–81% out of 100 pairs indicates "very large" difference between the groups, so does 19–20%. Notably, even if the thresholds in Table 1 are based on empirical datasets, when it comes to small, medium, and large effect sizes, the suggestions for *PHD* and *PHG* as well as *D* and *G* are very close to those by Vargha & Delaney (2000) given for their index of effect size, Vargha−Delaney *A* (see Metsämuuronen, 2025).

Table 1. Thresholds for estimates of effect sizes for binary items (Metsämuuronen, 2025)

	Cohen's d[1]	PHD[3]	PHG[3]	Somers' D[3]	Goodman–Kruskal G[3]
small	0.2	0.57 (0.43)	0.57 (0.43)	0.13	0.14
medium	0.5	0.65 (0.35)	0.65 (0.35)	0.29	0.31
large	0.8	0.72 (0.28)	0.73 (0.27)	0.43	0.45
very large	1.2[2]	0.80 (0.20)	0.81 (0.19)	0.59	0.62
huge	2[2]	0.91 (0.09)	0.93 (0.07)	0.81	0.84

1) Cohen (1969, 1988)
2) Sawilowsky (2009)
3) based on n = 7,948 estimates from empirical datasets by Metsämuuronen (2022f, 2023)

References

Berry, K. J., Johnston, J. E., & Mielke, P. W., Jr. (2018). *The measurement of correlation. A permutation statistical approach*. Springer. https://doi.org/10.1007/978-3-319-98926-6

Cohen, J. (1969). *Statistical power analysis for the behavioral sciences*. First Edition. Academic press.

Cohen, J. (1988). *Statistical power analysis for the behavioral sciences*. Second Edition. Erlbaum.

Cureton, E. E. (1956). Rank–biserial correlation. *Psychometrika*, *21*(3), 287–290. https://doi.org/10.1007%2FBF02289138

Goodman, L. A., & Kruskal, W. H. (1954). Measures of association for cross classifications. *Journal of the American Statistical Association*, *49*(268), 732–764. http://dx.doi.org/10.1080/01621459.1954.10501231

Higham, P. A., & Higham D. P. (2019). New improved gamma: Enhancing the accuracy of Goodman-Kruskal's gamma using ROC curves. *Behaviour Research Methods*, *51*(1), 108–125. http://dx.doi.org/10.3758/s13428-018-1125-5

IBM (2017). IBM SPSS Statistics 25 Algorithms. IBM. Retrieved from https://www.ibm.com/docs/en/SSLVMB_25.0.0/pdf/en/IBM_SPSS_Statistics_Algorithms.pdf (accessed Jan. 21, 2025).

Jonckheere, A. R. (1954). A distribution-free k–sample test against ordered alternatives. *Biometrika*, *41*(1–2), 133–145. http://dx.doi.org/10.1093/biomet/41.1-2.133

Kendall, M. G. *(*1938). A new measure of rank correlation. *Biometrika, 30*(1/2), 81–93. http://dx.doi.org/10.2307/2332226

Kendall, M. G. (1948). *Rank correlation methods.* First Edition. Charles Griffin & Co Ltd.

Kendall, M. G., & Gibbons, J. D. (1990). *Rank correlation methods.* Fifth Edition. Oxford University Press.

Kerby, D. S. (2014). The simple difference formula: An approach to teaching nonparametric correlation. *Comprehensive Psychology*, *3*, article 1. http://dx.doi.org/10.2466/11.IT.3.1

Kvålseth, T. O. (2017). An alternative measure of ordinal association as a value-validity correction of the Goodman–Kruskal gamma. *Communications in Statistics - Theory and Methods*, *46*(21), 10582–10593. http://dx.doi.org/10.1080/03610926.2016.1239114

Mann, H. B., & Whitney, D. R. (1947). On a test of whether one of two random variables is stochastically larger than the other. *Annals of Mathematical Statistics*, *18*(1), 50–60. http://dx.doi.org/10.1214/aoms/1177730491

Masson, M. E. J., & Rotello, C. M. (2009). Sources of bias in the Goodman–Kruskal gamma coefficient measure of association: Implications for studies of metacognitive processes. *Journal of Experimental Psychology: Learning, Memory, and Cognition*, *35*(2), 509–527. http://dx.doi.org/10.1037/a0014876

Metsämuuronen, J. (2020a). Somers' *D* as an alternative for the item–test and item–rest correlation coefficients in the educational measurement settings. *International Journal of Educational Methodology*, *6*(1), 207–221. https://doi.org/10.12973/ijem.6.1.207
Metsämuuronen, J. (2020b). Dimension-corrected Somers' D for the item analysis settings. *International Journal of Educational Methodology*, *6*(2), 297–317. https://doi.org/10.12973/ijem.6.2.297
Metsämuuronen J. (2021a). Goodman–Kruskal gamma and dimension-corrected gamma in educational measurement settings. *International Journal of Educational Methodology*, *7*(1), 95–118. https://doi.org/10.12973/ijem.7.1.95
Metsämuuronen, J. (2021b). Directional nature of Goodman-Kruskal gamma and some consequences. Identity of Goodman-Kruskal gamma and Somers' delta, and their connection to Jonckheere-Terpstra test statistic. *Behaviormetrika*, *48*(2). http://dx.doi.org/10.1007/s41237-021-00138-8
Metsämuuronen, J. (2022e). Rank–polyserial correlation: Quest for a "missing" coefficient of correlation. *Frontiers in Applied Mathematics and Statistics*, *8*:914932. http://dx.doi.org/10.3389/fams.2022.914932
Metsämuuronen, J. (2022f). Effect of various simultaneous sources of mechanical error in the estimators of correlation causing deflation in reliability. Seeking the best options of correlation for deflation-corrected reliability. *Behaviormetrika*, *49*(1), 91–130 https://doi.org/10.1007/s41237-022-00158-y
Metsämuuronen, J. (2023). Note on the radical deflation in t-test statistic, some consequences, and deflation- corrected t-test statistic. Preprint at http://dx.doi.org/10.13140/RG.2.2.25033.62564 (accessed Jan. 21, 2025).
Metsämuuronen, J. (2025). Five new common language estimators of effect size. *Journal of Experimental Education*, http://dx.doi.org/10.1080/00220973.2025.2459411
Newson, R. (2006). Confidence intervals for rank statistics: Somers' D and extensions. *The Stata Journal*, *6*(3), 309–334. http://www.stata-journal.com/sjpdf.html?articlenum=snp15_6
Newson, R. (2008). Identity of Somers' *D* and the rank biserial correlation coefficient. http://www.rogernewsonresources.org.uk/miscdocs/ranksum1.pdf (accessed Jan. 21, 2025).
Sawilowsky, S. (2009). New effect size rules of thumb. *Journal of Modern Applied Statistical Methods, 8*(2), 467–474. http://dx.doi.org/10.22237/jmasm/1257035100
Sheskin, D. J. (2011). *Handbook of parametric and nonparametric statistical procedures*. Fifth Edition. Chapman & Hall/CRC.
Siegel, S., & Castellan, N.J., Jr. (1988). *Nonparametric statistics for the behavioral sciences*. Second Edition. McGraw-Hill.
Sirkin, M. R. (2006). *Statistics of the social science.* Third Edition. SAGE Publications.
Somers, R. H. (1962). A new asymmetric measure of association for ordinal variables. *American Sociological Review*, *27*(6), 799–811. http://dx.doi.org/10.2307/2090408
Terpstra, T.J. (1952). The asymptotic normality and consistency of Kendall's test against trend, when ties are present in one ranking. *Indagationes Mathematicae, 14*(3), 327–333. http://dx.doi.org/10.1016/S1385-7258(52)50043-X
Van der Ark, L. A., & Van Aert, R. C. M. (2015). Comparing confidence intervals for Goodman and Kruskal's gamma coefficient. *Journal of Statistical Computation and Simulation*, *85*(12), 2491–2505. http://dx.doi.org/10.1080/00949655.2014.932791
Vargha, A. & Delaney, H. D. (2000) A critique and improvement of the CL common language effect size statistics of McGraw and Wong. *Journal of Educational and Behavioral Statistics*, *25*(2),101–132. https://doi.org/10.3102/10769986025002101
Wendt, H. W. (1972). Dealing with a common problem in social science: A simplified rank biserial coefficient of correlation based on the U statistic. *European Journal of Social Psychology*, *2*(4), 463–465. https://doi.org/10.1002/ejsp.2420020412

Wholey, J., S., Hatry, H., P., & Newcomer, K. E. (Eds.) (2015). *Handbook of practical program evaluation.* Fourth Edition. Jossey-Bass.

Wilcoxon, F. (1945). Individual comparisons by ranking methods. *Biometrics Bulletin*, *1*(6), 80–83. http://dx.doi.org/10.2307/3001968

Woods, C. M. (2007). Confidence intervals for gamma-family measures of ordinal association. *Psychological Methods, 12*(2), 185–204. http://dx.doi.org/10.1037/1082-989X.12.2.185

Supplementary Appendix 2

Formulae of common language estimators of reliability (CLER) based on alpha, theta, omega, and rho

In the main text, mainly the DCERs based on omega are discussed. Here, the formulae based on alpha, theta, and rho are given also. Also, some alternative forms based on means of *D*s and *G*s are given.

Traditional estimators of reliability

Estimators of reliability of the test score are many. The most used estimators are coefficients alpha, theta, omega, and rho (maximal reliability). Coefficient alpha (chronologically, Kuder and Richardson, 1937; Guttman, 1945, Cronbach, 1951) can be expressed as follows:

$$\rho_\alpha = \frac{k}{k-1}\left(1-\frac{\sum_{i=1}^{k}\sigma_i^2}{\left(\sum_{i=1}^{k}\sigma_i \times \rho_{iX}\right)^2}\right) \tag{1}$$

(Lord, Novick & Birnbaum, 1968), where σ_i^2 refers to the item variance and ρ_{iX} to the item–score correlation, that is, product–moment coefficient of correlation (PMC). Coefficients theta (chronologically, Lord, 1958; Kaiser & Caffrey, 1965; Armor, 1974) also known as Armor's theta can be expressed as follows:

$$\rho_{TH} = \frac{k}{k-1}\left(1-\frac{1}{\sum_{i=1}^{k}\lambda_{i\theta}^2}\right). \tag{2}$$

where $\lambda_{i\theta}^2$ is the square of the principal component loadings of the first or only principal component, that is, PMC between items and the principal component score. Coefficient omega (Heise & Bohrnstedt, 1970; McDonald, 1970, 1999), also known as McDonald's omega or omega total, can be expressed as follows:

$$\rho_\omega = \frac{\left(\sum_{i=1}^{k}\lambda_{i\theta}\right)^2}{\left(\sum_{i=1}^{k}\lambda_{i\theta}\right)^2+\sum_{i=1}^{k}\left(1-\lambda_{i\theta}^2\right)}, \tag{3}$$

where $\lambda_{i\theta}$ is the factor loadings of the one-factor model, that is, PMC between items and the factor score variable. Coefficient rho or maximal reliability (e.g., Li, 1997; Li, Rosenthal, & Rubin, 1996; Raykov, 1997, 2004) also known as Raykov's rho and which is equivalent with Hancock's H can be expressed as follows:

$$\rho_{MAX} = \frac{1}{1+\frac{1}{\sum_{i=1}^{k}\left(\lambda_{i\theta}^2/\left(1-\lambda_{i\theta}^2\right)\right)}}. \tag{4}$$

Of the estimators, Eq. (4) may produce overestimates with small or finite sample sizes (see Aquirre-Urreta, Rönkkö, & McIntosh, 2019; Metsämuuronen, 2022a, 2022b). The reason is that the element $\lambda_{i\theta}^2/\left(1-\lambda_{i\theta}^2\right)$ is instable if the magnitude of any of the item–score correlations is very high, and ρ_{MAX} is obviously not defined when $\lambda_{i\theta}$ = 1. These kinds of extreme conditions may easily happen with small sample sizes. These estimators are used as bases for the deflation-corrected estimators of reliability (DCER). In DCERs, the deflation-prone estimator of correlation, PMC, is replaced by more stable estimators or association as the linking element between the items and the score variable.

Metsämuuronen (2022a, 2022d, 2022g) points that using theta, omega, and rho outside of their traditional context of principal component and factor analysis is debatable. Within the paradigm related to DCERs, however, it is assumed that these estimators *could* be used as independent estimators. Alternatively, it is possible to think that the estimates by using *G* or *D* instead of the traditional $\lambda_{i\theta}$ could be outcomes of renewed procedures on principal component and factor analysis (cl. ordinal theta by Zumbo, Gadermann, & Zeisser, 2007). It is good to note, again, the possible challenge in the forms based on rho (Eqs. 24a and 24b) not to be defined when *D* and *G* reaches the value 1 in any of the items. This condition easily occurs with small sample sizes where all test takers may get a unique score.

DCERs using D and G as the linking coefficient

By replacing ρ_{iX} and $\lambda_{i\theta}$ in Eqs. (1) to (4) by Somers' D (Somers, 1962) or Goodman–Kruskal *G* (Goodman & Kruskal, 1954), we get a variety of deflation-corrected estimators of reliability based on different forms of reliability. Estimators "*alphaD*" and "*alphaG*" are based on the form of coefficient alpha as follows:

$$\rho_{\alpha\_D} = \frac{k}{k-1}\left(1-\frac{\sum_{i=1}^{k}\sigma_i^2}{\left(\sum_{i=1}^{k}\sigma_i \times D_i\right)^2}\right) \tag{5a}$$

and

$$\rho_{\alpha\_G} = \frac{k}{k-1}\left(1-\frac{\sum_{i=1}^{k}\sigma_i^2}{\left(\sum_{i=1}^{k}\sigma_i \times G_i\right)^2}\right), \tag{5b}$$

where the notations *α_D* and *α_G* indicate that the base of the coefficient is alpha and the weight factor w_i is either *D* or *G*. Similarly, we get "*thetaD*" and "*thetaG*" as follows:

$$\rho_{TH\_D} = \frac{k}{k-1}\left(1-\frac{1}{\sum_{i=1}^{k} D_i^2}\right) \tag{6a}$$

and

$$\rho_{TH\_G} = \frac{k}{k-1}\left(1-\frac{1}{\sum_{i=1}^{k} G_i^2}\right), \tag{6b}$$

"*omegaD*" and "*omegaG*" as follows:

$$\rho_{\omega\_D} = \frac{\left(\sum_{i=1}^{k} D_i\right)^2}{\left(\sum_{i=1}^{k} D_i\right)^2 + \sum_{i=1}^{k}\left(1-D_i^2\right)} \tag{7a}$$

and

$$\rho_{\omega\_G} = \frac{\left(\sum_{i=1}^{k} G_i\right)^2}{\left(\sum_{i=1}^{k} G_i\right)^2 + \sum_{i=1}^{k}\left(1-G_i^2\right)}, \tag{7b}$$

and "*rhoD*" and "*rhoG*" as follows:

$$\rho_{MAX\_D} = \frac{1}{1+\frac{1}{\sum_{i=1}^{k}\left(D_i^2/\left(1-D_i^2\right)\right)}} \tag{8a}$$

and

$$\rho_{MAX\_G} = \frac{1}{1+\frac{1}{\sum_{i=1}^{k}\left(G_i^2/\left(1-G_i^2\right)\right)}}. \tag{8b}$$

Alternatively, with estimators based on theta and omega, algebraically identical result is obtained if, instead of sums of the item–score associations, the *average* values are used multiplied by *k*. Hence, as an example, Eq. (6a) can be expressed as $\rho_{TH\_D} = \frac{k}{k-1}\left(1-\left(k\times\bar{D}^2\right)^{-1}\right)$, where $\bar{D}^2$ refers to the mean of all D_i^2 over all items. Of the estimators, those using *D* as the weight factor are more conservative than those using *G*. Estimators based on alpha, theta, and omega are conservative and those based on rho are liberal, that is, with small or finite sample size sizes the estimates by alpha, theta, and omega tend to underestimate the population value mildly and those by rho tend to give mild overestimates (see Metsämuuronen, 2022a).

Common language estimators of reliability

From the common language viewpoint, the interesting form of *D* is as follows:

$$D(g|X) = D = 2 \times \frac{JT}{\sum_{l<h}^{R} n_l n_h} - 1 = 2 \times PHD - 1 \qquad (9)$$

where *JT* refers to the Jonckheere–Terpstra test statistics and n_i and n_j are the sample sizes in sub-populations $i < j$ (see Appendix 1). The parallel form related to *G* is as follows:

$$G(g|X) = G = 2 \times \frac{JT}{\sum_{l<h}^{R} (n_l n_h) - T} - 1 = 2 \times PHG - 1 \qquad (10)$$

where T refers to the number of tied pairs between the variable. The element $JT \Big/ \sum_{l<h}^{R} n_l n_h = PHD$ in Eq. (9) and, $JT \Big/ \left(\sum_{l<h}^{R} (n_l n_h) - T \right) = PHG$ in Eq. (10) strictly refer to the proportion of test takers from the higher subpopulation(s) who score higher in the total test in comparison with those who came from the lower subpopulation(s).

When it comes to the estimators of reliability, the sum of items is of interest. Then, either the sum of *D*s or *G*s (Eqs. 5a to 6b; in alpha and omega) or the sum of squares (Eqs. 6a to 8b; in omega, theta, and rho) is of interest. When considering the sum of items, we can transform Eq. (9) in the following form:

$$\sum_{i=1}^{k} D_i = \sum_{i=1}^{k} \left(2 \times \frac{JT_i}{\sum_{l<h}^{R_i} n_l n_h} - 1 \right) = 2 \times \sum_{i=1}^{k} \left(\frac{JT_i}{\sum_{l<h}^{R_i} n_l n_h} \right) - k = 2 \times \sum_{i=1}^{k} (PHD_i) - k \,, \qquad (11)$$

and when the sum of squares is considered, we get the form as follows:

$$\sum_{i=1}^{k} D_i^2 = \sum_{i=1}^{k} \left(2 \times \frac{JT_i}{\sum_{l<h}^{R_i} n_l n_h} - 1 \right)^2 = \sum_{i=1}^{k} (2 \times PHD_i - 1)^2 = 4 \times \left(\sum_{i=1}^{k} PHD_i^2 - \sum_{i=1}^{k} PHD_i \right) + k \,, \qquad (12)$$

where $\sum_{i=1}^{k} PHD_i^2$ refers to the sum of squared *PHD*s over all items. Alternatively, instead of the sums of *PHD*s, we can use the *mean* of *PHD*s (*MPHD*):

$$MPHD = \sum_{i=1}^{k} PHD_i \Big/ k \,). \qquad (13)$$

Then, Eq. (11) gets the form

$$\sum_{i=1}^{k} D_i = k \times (2 \times MPHD - 1) \tag{14}$$

and Eq. (12) the form

$$\sum_{i=1}^{k} D_i^2 = k \times \left(4 \times \left(MPHD^2 - MPHD\right) + 1\right), \tag{15}$$

where $MPHD^2$ is the mean of squared *PHD*s. The corresponding forms related to *G* equal with Eqs. (11) and (12) expect that the element $\sum_{l<h}^{R_i} n_l n_h$ is $\sum_{l<h}^{R_i} (n_l n_h) - T$, and the corresponding forms using the means are denoted by *MPHG* and $MPHG^2$. In both cases, the elements *PHD* = $\sum_{i=1}^{k} \left(JT_i \Big/ \sum_{l<h}^{R_i} n_l n_h \right)$ and *PHG* = $\sum_{i=1}^{k} \left(JT_i \Big/ \left(\sum_{l<h}^{R_i} (n_l n_h) - T \right) \right)$ refer to the *sum* of proportions of higher group dominance over all the items in the compilation while *MPHD* and *MPHG* refer to the *average* proportions of higher group dominance over all the items in the compilation.

From the common language viewpoint, the "perfect" reliability (*REL* = 1) gets a specific meaning. *REL* = 1 refers to the pattern where *all test takers* from the higher subpopulation(s) in *all items* scored higher in the total score than the test takers from the lower subpopulation(s). Specifically, in the binary case, the perfect reliability refers to a pattern where in all items all test takers who gave the correct answer in the item scored higher in the test than those who gave an incorrect answer. With *G* in Eqs. (27) and (28), the interpretation is similar even *without* the extension *if we consider only those cases with which the total score was not identical between the test takers with different item score* because the pattern that simultaneously *D* = *G* = 1 indicates that there are no tied pairs since the condition of *D* = *G* is possible only in the case that there are no tied pairs, i.e., when *T* in Eq. (10) equals *T* = 0.

From the technical perspective, with deterministically discriminating set of items, the observed *JT* statistic reaches the maximal possible value in each item, and this leads to

$$JT_i = \sum_{l<h}^{R_i} (n_l n_h) - T = \sum_{l<h}^{R_i} n_l n_h \tag{16}$$

and, consequently, to *D* = *G* = 1 and to

$$\sum_{i=1}^{k} \left(JT_i \Big/ \left(\sum_{l<h}^{R_i} (n_l n_h) - T \right) \right) = \sum_{i=1}^{k} \left(JT_i \Big/ \sum_{l<h}^{R_i} n_l n_h \right) = k\,. \tag{17}$$

Then, because of Eq. (11),

$$\sum_{i=1}^{k} D_i = \sum_{i=1}^{k} G_i = 2 \times k - k = k \tag{18}$$

and, because of Eq. (12),

$$\sum_{i=1}^{k} D_i^2 = \sum_{i=1}^{k} G_i^2 = \sum_{i=1}^{k} (2 \times 1 - 1)^2 = k\,. \tag{19}$$

Consequently, the estimates of reliability by *thetaD, thetaG, omegaD,* and *omegaG* are as follows: $\rho_{TH\_D} = \rho_{TH\_G} = \frac{k}{k-1}\left(1-\frac{1}{k}\right) \equiv 1$ and $\rho_{\omega\_D} = \rho_{\omega\_G} = \frac{k^2}{k^2+0} \equiv 1$.

In the deterministic case, *rhoD* and *rhoG* are not defined, and *alphaD* and *alphaG* underestimate reliability mildly:

$$\rho_{\alpha\_Di\theta} = \rho_{\alpha\_Gi\theta} = \frac{k}{k-1}\left(1-\sum_{i=1}^{k}\sigma_i^2 \Big/ \left(\sum_{i=1}^{k}\sigma_i\right)^2\right) < 1. \tag{20}$$

Only in the condition that $D = G = 1$ *and* the item variances are equal ($\sigma_i^2 = \sigma_j^2 = \sigma^2$), e.g., when the items are standardized, alpha reaches the "perfect" reliability:

$$\rho_{\alpha\_Di\theta} = \rho_{\alpha\_Gi\theta} = \frac{k}{k-1}\left(1-\frac{k\sigma^2}{k^2\sigma^2}\right) \equiv 1. \tag{21}$$

Notably, if all items are standardized, the form of alpha equals the form of theta. We may note that, in the case of deterministic discrimination, the traditional alpha can reach the value *REL* = 1 only in the special (theoretical) case that all the item difficulties and item variances are identical. This specific pattern in the data set leads to the condition where the number of categories in the items equals the number of categories in the score. This is the only condition where $\sigma_i^2 = \sigma_j^2 = \sigma^2$ and *Rit* = 1 leading to *REL* = 1.

In the general case, the forms of DCERs related to *D* and *G,* and showing the common language element, can be expressed as follows. Because of Eqs. (5a) and (11), the CLERs based on alpha can be expressed as follows:

$$\rho_{\alpha\_D} = \frac{k}{k-1}\left(1-\sum_{i=1}^{k}\sigma_i^2 \Big/ \left(\sum_{i=1}^{k}\sigma_i \times \left(2\times PHD_i - 1\right)\right)^2\right) \tag{22a}$$

and, because of Eqs. (5b) and (11),

$$\rho_{\alpha\_G} = \frac{k}{k-1}\left(1-\sum_{i=1}^{k}\sigma_i^2 \Big/ \left(\sum_{i=1}^{k}\sigma_i \times \left(2\times PHG_i - 1\right)\right)^2\right). \tag{22b}$$

Because of Eqs. (6a) and (11), when using *D* as the weight factor, CLER based on theta (*thetaD*) can be expressed as follows:

$$\rho_{TH\_D} = \frac{k}{k-1}\left(1-\left(\sum_{i=1}^{k}\left(2\times PHD_i - 1\right)^2\right)^{-1}\right) \tag{23a}$$

or, if the mean of *PHD*s is used, because of Eqs. (6a) and (14),

$$\rho_{TH\_D} = \frac{k}{k-1}\left(1-\left(k\times\left(4\times\left(MPHD^2 - MPHD\right)+1\right)\right)^{-1}\right). \tag{23b}$$

Because of Eqs. (6b) and (12), when using *G* as the weight factor, CLER based on theta (*thetaG*) can be expressed as follows:

$$\rho_{TH\_G} = \frac{k}{k-1}\left(1-\sum_{i=1}^{k}\left(2\times PHG_i-1\right)^{-2}\right) \tag{24a}$$

or, if the mean of *PHG*s is used, because of Eqs. (6b) and (15),

$$\rho_{TH\_G} = \frac{k}{k-1}\left(1-\left(k\times\left(4\times\left(MPHG^2-MPHG\right)+1\right)\right)^{-1}\right). \tag{24b}$$

Correspondingly, because of Eqs. (7a) and (14), CLER based on omega and using *D* as the weight factor (*omegaD*) can be expressed as follows:

$$\rho_{\omega\_D} = \frac{\left(\sum_{i=1}^{k}\left(2\times PHD_i-1\right)\right)^2}{\left(\sum_{i=1}^{k}\left(2\times PHD_i-1\right)\right)^2+\sum_{i=1}^{k}\left(1-\left(2\times PHD_i-1\right)^2\right)} \tag{25a}$$

or, if the means of *PHD*s are used, because of Eqs. (7a) and (15),

$$\rho_{\omega\_D} = \frac{k\times\left(2\times MPHD-1\right)^2}{k\times\left(2\times MPHD-1\right)^2+k\times\left[1-\left(4\times\left(MPHD^2-MPHD\right)+1\right)\right]}\ . \tag{25b}$$

Because of Eqs. (7b) and (14), CLER based on omega and using *G* as the weight factor (*omegaG*) can be expressed as follows:

$$\rho_{\omega\_G} = \frac{\left(\sum_{i=1}^{k}\left(2\times PHG_i-1\right)\right)^2}{\left(\sum_{i=1}^{k}\left(2\times PHG_i-1\right)\right)^2+\sum_{i=1}^{k}\left(1-\left(2\times PHG_i-1\right)^2\right)} \tag{26a}$$

or, if the means of *PHG*s are used, because of Eqs. (7b) and (15),

$$\rho_{\omega\_G} = \frac{k\times\left(2\times MPHG-1\right)^2}{k\times\left(2\times MPHG-1\right)^2+k\times\left[1-\left(4\times\left(MPHG^2-MPHG\right)+1\right)\right]}. \tag{26b}$$

Finally, because of Eqs. (8a) and (14), CLER based on rho and using *D* as the weight factor (*rhoD*), can be expressed as follows:

$$\rho_{MAX\_D} = \frac{1}{1+\frac{1}{\sum_{i=1}^{k}\left(\left(2\times PHD_i-1\right)^2/\left(1-\left(2\times PHD_i-1\right)^2\right)\right)}} \tag{27a}$$

and, because of Eqs. (8b) and (14),

$$\rho_{MAX\_G} = \frac{1}{1+\frac{1}{\sum_{i=1}^{k}\left(\left(2\times PHG_i-1\right)^2/\left(1-\left(2\times PHG_i-1\right)^2\right)\right)}}. \tag{27b}$$

Although it may be possible to express the estimators based on rho by means of *PHD* and *PHG*, they are unnecessarily complicated to show here. Again, we may note that the estimators based on rho are not suggested to be used with small sample sizes because, unlike *Rit* which never reaches the limits of correlation with item and score in the real-life settings, *D* and *G* easily reach the extreme value of correlation (±1) with small samples. The formulae are not very practical in common use because far more easily the outcomes plainly by using *D* and *G* in the forms. The forms are derived just to show the elements of the common language effect size embedded in the formulae.

References

Aquirre-Urreta, M., Rönkkö, M., & McIntosh, C. N. (2019). A Cautionary note on the finite sample behavior of maximal reliability. *Psychological Methods*. *24*(2), 236–252. https://doi.org/10.1037/met0000176

Armor, D. (1973). Theta reliability and factor scaling. *Sociological Methodology*, *5*, 17–50. https://doi.org/10.2307/270831

Cronbach, L. J. (1951). Coefficient alpha and the internal structure of tests. *Psychometrika*, *16*(3) 297–334. https://doi.org/10.1007/BF02310555

Goodman, L. A., & Kruskal, W. H. (1954). Measures of association for cross classifications. *Journal of the American Statistical Association*, *49*(268), 732–764. http://dx.doi.org/10.1080/01621459.1954.10501231

Guttman, L. (1945). A basis for analyzing test-retest reliability. *Psychometrika*, *10*(4), 255–282. https://doi.org/10.1007/BF02288892.

Heise, D., & Bohrnstedt, G. (1970). Validity, invalidity, and reliability. *Sociological Methodology*, *2*, 104–129. https://doi.org/10.2307/270785

Kaiser, H. F., & Caffrey, J. (1965). Alpha factor analysis. *Psychometrika*, *30*, 1−14. https://doi.org/10.1007/BF02289743

Kuder, G. F. & Richardson, M. W. (1937). The theory of the estimation of test reliability. *Psychometrika*, *2*(3), 151–160. http://dx.doi.org/10.1007/BF02288391

Li, H. (1997). A unifying expression for the maximal reliability of a linear composite. *Psychometrika*, *62*(2), 245–249. http://dx.doi.org/10.1007/BF02295278

Li, H., Rosenthal, R., & Rubin, D. B. (1996). Reliability of measurement in psychology: From Spearman-Brown to maximal reliability. *Psychological Methods*, *1*(1), 98–107. http://dx.doi.org/10.1037/1082-989X.1.1.98

Lord, F. M. (1958). Some relations between Guttman's principal component scale analysis and other psychometric theory. *Psychometrika*, *23*(4), 291–296. http://dx.doi.org/10.1002/j.2333-8504.1957.tb00073.x

Lord, F. M., Novick, M. R., & Birnbaum, A. (1968). *Statistical theories of mental test scores*. Addison–Wesley Publishing Company.

Metsämuuronen, J. (2022a). Typology of deflation-corrected estimators of reliability. *Frontiers in Psychology*, *13*:891959. http://dx.doi.org/10.3389/fpsyg.2022.891959

Metsämuuronen, J. (2022b). How to obtain the most error-free estimate of reliability? Eight sources of underestimation of reliability. *Practical Assessment, Research, and Evaluation, PARE*, *27*(1), Art. 10. https://doi.org/10.7275/7nkb-j673

Metsämuuronen, J. (2022d). Deflation-corrected estimators of reliability. *Frontiers in Psychology*, *12*:748672, https://doi.org/10.3389/fpsyg.2021.748672

Metsämuuronen, J. (2022g). Attenuation-corrected reliability and some other MEC-corrected estimators of reliability. *Applied Psychological Measurement*, https://doi.org/10.1177/01466216221108131

Raykov, T. (1997). Estimation of composite reliability for congeneric measures. *Applied Psychological Measurement*, *21*(2), 173–184. https://doi.org/10.1177/01466216970212006

Raykov, T. (2004). Estimation of maximal reliability: A note on a covariance structure modeling approach. *British Journal of Mathematical and Statistical Psychology, 57*(1), 21–27. http://doi.org/10.1348/000711004849295

Zumbo, B. D., Gadermann, A. M., & Zeisser, C. (2007). Ordinal versions of coefficients alpha and theta for Likert rating scales. *Journal of Modern Applied Statistical Methods, 6*(1), 21–29. http://dx.doi.org/10.22237/jmasm/1177992180
